# Long Term Remission of Anorexia Nervosa: Factors Involved in the Outcome of Female Patients

**DOI:** 10.1371/journal.pone.0056275

**Published:** 2013-02-27

**Authors:** Cybele R. Espíndola, Sergio L. Blay

**Affiliations:** Department of Psychiatry, Federal University of São Paulo (Escola Paulista de Medicina - UNIFESP), São Paulo, Brazil; The University of Queensland, Australia

## Abstract

**Background:**

Anorexia nervosa (AN) is usually marked by difficult recovery.

**Objective:**

To elicit, describe and characterize factors involved in successful AN remission for at least five years and post-recovery life.

**Methods:**

A qualitative study design using semi-structured interviews according to grounded theory methodology was used. An intentional sample of 15 information rich AN patients was selected using two sampling procedures: the criterion and “snowball” techniques. Qualitative interviews were audiotaped, transcribed, and entered into a content analysis. Researchers coded transcripts and developed themes.

**Results:**

Four core parameters were found to be associated with remission: (i) ‘motivation to change/stimuli’; (ii) ‘empowerment/autonomy’; (iii) ‘media related factors’; (iv) ‘treatment factors’. Clinical remission can be associated with residual symptoms.

**Conclusions:**

The recovery model involves not only one, but rather a set of inter-active variables, each one can partially explain remission. Media factors can take a new role on recovery. Remission, however, can be followed by remnants of the disease and functional limitations.

## Introduction

Anorexia nervosa is relatively rare among the general population and rather common among young women [1]. It is manifested by symptoms leading to significant clinical impairment and distress [2]. Severe cases need hospitalization and mortality is important in this group. In a meta-analysis of excess mortality, anorexia nervosa was associated with the highest rate of mortality of all mental disorders [3].

A growing body of literature is accumulating on the course of AN. Some reflect optimism [4] generally in early onset and short history group [5] but others, mostly treatment-outcome studies, have shown association with chronicity [6], [7]; reluctance to recover [8]; poor adherence [9]; impulsivity and severity [10]; comorbidity [11]; and mortality [11]–[20].

Studies with qualitative designs on patient recovery after treatment for AN provide some potentially useful insights and have shown that several internal and external factors have an important impact on outcome. A recent systematic review and metasynthesis found that the outcome may be affected by internal motivation to change, religion and spirituality; the perceived value of the treatment experience; developing supportive relationships; awareness and tolerance of negative emotion; relevant turning points; fear of change; therapeutic relationships; sound disorders' information among other factors [21].

Findings from studies focusing on AN, particularly those relating to the impact of treatment on women's experiences of remission on the short term follow-up may be only partly applicable to women in long term follow-up. To date there has been little published research describing the factors involved in the long term remission in women with AN [22]–[27]. Nilsson and Hägglöf [27] moved a step further in this area by investigating if the recovery process was distinguished by some “turning-points.” Major findings indicated that family, friends, boyfriends, personal decisions, activities and treatment are all key factors with remission.

To our knowledge there has been little published research describing experiences of remission in women with AN, or their views over alternative treatments, general AN information, media related factors and life after remission. In order to fill this gap, we carried out a qualitative study to exam the inner experiences and external factors associated with young women with AN in remission for at least five years.

## Methods

### Study Design

Ethnographic interviewing elicited information from women with AN in remission for at least 5 years. Grounded theory, a method of qualitative analysis [28] was used to elucidate the perception of the interviewees revealed in the narrative data [29].

Purposeful samples, with information-rich cases, comprise the sample, considering the patients as experts and interviewing them accordingly. In this study, two strategies were employed. First, criterion sampling in which the following criteria were used: 1- women who had SCID/DSM-IV anorexia nervosa and, 2- remission for at least five years. In this study, remission was considered as the absence of symptoms, which does not meet the DSM-IV criteria, and confirmed simultaneously by three people: the patient's self-report; the assistant doctor; and a relevant family member. The assistant doctor was blinded, in other words, not involved with data collection, data analysis or writing the manuscript. Individuals in the sample were selected with the aid of their medical doctors specialized in the treatment of eating disorders. Potential informants were told about the study by their doctors. They were provided with written information about the study. All participants gave their written consent. The second strategy involved “snowball”, in which selected participants could suggest other ‘information rich’ subjects [30]. Patients with acute psychotic symptoms, mental impairment, cognitive deficits or a certain speech or auditory impairment that could compromise communication with the researcher were not included. In addition, individuals with alcohol or drug abuse or dependence were not included if acutely intoxicated.

Socioeconomic data were obtained through [31], allowing classification into five classes, from A (individuals with the highest income level: above 15 minimum wages/month [US$ 4,030.00]) to E (those with the lowest income level: equal to or below ½ minimum wage/month, [US$ 134.00]).

### Interview Technique

To elicit the women's experiences on this sensitive topic we used semistructured, face-to-face interviews [32]. The interviews focused on experiences, information, feelings and personal opinions associated with AN remission. The interviewer was an experienced staff member (C.R.E.) who had not treated the subjects they interviewed. Training included the examination and discussion of six patients with different eating disorders at the outpatient clinic.

The interviews were tape-recorded with high quality equipment and immediately transcribed verbatim. All participants were interviewed in a private location. Each interview was voluntary and lasted between 90 and 120 minutes. For this study about the patients' subjective view on the remission of their AN, the following questions were used: ‘How did your eating disorder begin?’; ‘What contributed to a favorable evolution and recovery?’; ‘What helped and hindered you the most throughout the process?’; ‘What was your life like after recovery?’. Field notes were made to record events and perceptions throughout evaluation.

The sample size was defined by criteria of data saturation [30], [33]. The number of subjects was achieved when no new information was being added. When 15 participants were assessed, the criteria of data saturation were fulfilled.

### Analysis

Authors (C.E.; S.L.B.) independently reviewed transcripts to identify common themes which were developed into a preliminary coding scheme. Frequent group discussions helped increase agreement with the coding system and led to the development of a thematic structure, including both manifest (explicit) and latent themes. Coding was compared and differences of opinion resolved through examination of the text. Cohen's Kappa interrater reliability ranged from .70 to 1.0 for each thematic item. All constructs were validated against the original text using confirmatory and selective coding and following the ‘top-down principle’ [33]. Data was collected after approval by the Ethics Committee (1468/08), of the Federal University of São Paulo.

## Results

Fifteen women with remitted AN were interviewed between November 2008 and May 2009. Most of the participants were young women, with early onset AN (15 to 24 years old), were mostly single or divorced, with a high education and socioeconomic level. One participant achieved remission for ten years. See table 1.

**Table 1:** Socio-demographic and time in remission of participants.

Participant	Age	Employment	Level of education	Religious affiliation	Sec*	Marital status	Children	Remission Years
1	23	Student Nutrition	Incomplete higher education	Catholic	B1	Single	No	6 ½
2	26	Public service	Secondary education	Catholic	B1	Married	1	5
3	26	Nurse	Postgraduate level	African religion	B1	Single	No	10
4	22	Student Physical Education	Incomplete higher education	Catholic	B2	Single	No	6 ½
5	28	Student Medicine	Incomplete higher education	Spiritualist	B2	Single	No	7/3 months
6	23	Student Nutrition	Incomplete higher education	Evangelical	B2	Single	No	6 ½
7	28	Student Nursing	Incomplete higher education	Evangelical	B2	Divorced	1	7/2 months
8	30	Student Psychology	Complete higher education	None	A1	Single	No	6
9	26	Student Law	Incomplete higher education	Catholic	A2	Single	No	5/5 months
10	29	Architect	Complete higher education	Catholic	A1	Married	1	6
11	24	Layer	Complete higher education	Evangelical	B2	Single	No	5 ½
12	26	Public service	Complete secondary education	Catholic	C1	Married	1	5
13	32	Psychologist	Complete higher education	Evangelical	B1	Divorced	No	9/3 months
14	31	Housewife	Complete secondary education	None	B2	Married	2	9
15	29	Hairdresser	Complete secondary education	Spiritualist	C1	Single	No	8 ½

* Sec, socioeconomic level.

All participants (n = 15) had undergone treatment with psychotropic medication (selective serotonin reuptake inhibitors); some had psychotropics plus psychotherapy (n = 12); some had combined treatment with a nutritionist (n = 10); some had alternative treatments combined with drugs or psychotherapy (n = 4). Three patients needed hospitalization. Search for treatment occurred between 6 to 18 months of the index episode.

Participant's descriptions of their remission experience revealed several preliminary categories: personal factors; external factors; treatment factors. Each component has multiple dimensions. See table 2.

**Table 2:** Primary categories.

**Successful factors for remission**
*Personal factors*
1. Physical complications/imminence of death
2. Pregnancy
3. Willingness to change/determination
4. Self-acceptance
5. Autonomy in relation to family environment
6. Personal records (journals)
7. Spirituality
*External factors*
1. Affective relationships of support
2. Information about the disease from the media
*Treatment factors*
1. Multidisciplinary treatment
2. Hospital treatment
3. Drug treatment
4. Treatment with nutritionist
5. Psychotherapeutic treatment
6. Alternative therapies
**Difficulties associated with the process of remission (oppositive views)**
1. Change of habits
2. Ambiguity

By condensing the preliminary categories that contain a description of an experience that the informants identified as contributing to the remission process we were able to identify four major high order constructs. As we are willing to collect the participants own experiences associated with remission, these four higher order constructs are built on a bottom-up structure, in other words, this is a set of information brewed by the patients themselves. The following are the four constructs: 1) motivation and stimuli for remission; 2) empowerment/autonomy; 3) media related factors; and 4) treatment factors. See table 3.

**Table 3:** Secondary thematic categories.

Core factors of remission
**1. Motivation and stimuli to remission**
Desire to change/determination
Affective relationships of support
Pregnancy
Physical complications/imminence of death
**2. Empowerment/Autonomy**
Autonomy in relation to family environment
Self-acceptance
Spirituality
**3. Media related factors**
Personal records, journals, magazines
Internet
Information about the disease from the media
Conferences
**4.Treatment factors**
Multidisciplinary treatment
Hospital treatment
Drug treatment
Treatment with nutritionist
Psychotherapeutic treatment
Alternative therapies

### Motivation and stimuli to remission

Recovery is a process that requires powerful determinants. A total of four factors associated with motivation and stimuli to change were identified: willingness to change/determination; affective relationships of support; pregnancy; and physical complications/imminence of death. These aspects were mentioned spontaneously by a great number of interviewees as a wake-up call to action as regards the need to change. This could arise from an inner personal process or be related to key external events. Some patients associated their change with the process of pregnancy. This event reorganized these women's lives by changing their eating pattern (“*I started eating everything as usual again… I hadn't eaten for about three years*” – I 2) or re-guiding them psychologically (“*…my focus changed, it wasn't my body anymore, but my baby's…*” – I 14). Imminence or risk of death was also mentioned as an important determinant for change to occur (“*risk of fractures… or infections*…” – I 4). One group of patients reported that interpersonal relationships with meaningful people such as parents or friends had a positive repercussion on change and recovery (“*My father either made or bought good food, those I liked best just to please me and attract me….” –* I 4).

### Empowerment/Autonomy

The ability to perceive and care for oneself was frequently pointed out by patients as relevant for remission. A total of three aspects were emphasized: autonomy in relation to family environment; self-acceptance; and spirituality. Family organization can work in an intrusive way, restricting the individual's autonomy and independence. The meaning of autonomy in relation to family, i.e. the distinction between what is personal life and what is family life can be illustrated by the following account, *“When I overcame my fear of speaking up, of saying ‘no’ and going against my family, I grew stronger and overcame anorexia… leaving my home and my parents being distant for a time, this was essential for my cure.” –* I 11). One interviewee indicated the relevance of accepting her personal characteristics, flaws, limitations and inner growth as determinants of a more integrated personal life (*“As I became more mature, I began to focus on other things… Then, I started dating and having a social life, which helped me in this process of acceptance” –* I 3). Spirituality was reported as a powerful instrument to help recovery (*“…I didn't feel alone at all, because I believed there was a higher power, stronger than all the ghosts, stronger than this disease.” –* I 7).

### Media-related factors

Personal records (diaries) were pointed out as important by a great number of patients. Writing one's thoughts and experiences seems to help the process without fear of external judgment. One patient reported, *“…my diary became my friend… I put all that tormented me in it, without the fear of being judged.” –* I 10.

The analysis of interviews in this study showed that individuals in the sample indicated several types of media as useful for remission. Journals, magazines, lectures and information on the internet were mentioned, among other things. Interviewee 11 reported the following, *“While I was sick, I attended a lecture and it felt like they were talking about me, it was kind of embarrassing, you know… and, that was the first moment I realized all that happens, the physiology of hunger, what goes on in the brain.”*

### Treatment factors

Several modalities of treatment contributed to remission. Among them, the following stood out: multidisciplinary, hospital, psychotherapeutic, drug and nutritional treatments and alternative therapies. Interviews showed the complexity of treatment, partly explaining the result, as illustrated by the following account, *“Anorexia can't be treated in a single way. There's no magic solution. Anything, however good it is, is not enough. So, you gotta try everything you can, all therapies and treatments in use. Only with all professionals working together and caring for each part of you at the same time can you pull it off…” –* I 14. In addition, interviewee 13 mentioned the following, *“Medication is useful, but it does nothing alone… it helps you feel less depressed, brings back a little hope and the will to try”*. Psychotherapy was emphasized, especially as regards its welcoming, empathetic and non-critical aspect, *“The therapist was the most important person during my recovery, because speaking to her about how I felt and what I thought about, and also feeling accepted by her, were the most healing aspects to me…”* – I 15. Psychoeducational interventions were found to be equally useful, since they consist of the transmission of information, such as the definition of relevant concepts of foods and the exemplification of patterns of hunger and food consumption, *“To know about nutrition and my body needs helped me to face the fear of eating some foods that I thought were dangerous.”* – I 15.

After treatment, and following a period of recovery, participants believed that remission appeared as the effect of alternative treatments, such as meditation and yoga, due to their capacity to change one's focus of attention and reduce anxiety.

Some interviewees emphasized great difficulties in the process of remission. One major obstacle found during treatment of anorexic patients is the fact that they begin treatment with little or almost no intention to progress. The need to change habits and interrupt restrictive practices cause fear and resistance, *“Because you gotta let go of everything you've been fighting for, your fear of growing fat and losing control… it's insane!…”* – I 1. Another aspect that should be emphasized is ambiguity in relation to the desire to change and the maintenance of the status quo.

Whereas some participants reported they felt well after remission, others showed that remnants of the disease were still present. Some interviewees mentioned these phenomena in more detail, *“Now, I have a healthy concern about my body, but it's very difficult to weigh myself, to get on the scale. I really don't like this, I avoid it whenever I can…” –* I 3. Another mentioned the following, *“It seems that anorexia is there across the street and any slip will make me get there, you know?”* – I 6.

When the women began to recover from AN, many began to take an interest in their past and present life style. Some had reached a point in their recovery where they felt swindled as they evaluated AN's negative impact in terms of personal development, such as difficulties in relationships, limitations, restrictions, inhibitions and loss of opportunities, among other aspects, as exemplified in the following account, *“My life got better, but I felt some things got lost on the way… Nowadays, I'm still single and don't have a boyfriend, while most people my age already do. Also, things could be better professionally speaking, if I didn't have this problem. It feels like I've missed the boat.”* – I 5.

## Discussion

After at least 5 years, all of the participants could vividly remember factors associated with their recovery process. In this study of women with AN and their experiences with remission we found four core factors involved with remission: ‘motivation and stimuli to remission’ when the desire to change and powerful other factors such as pregnancy or imminence of death triggers the process; ‘empowerment/autonomy’ when remission seems possible through a sense of autonomy, self acceptance and increased involvement with religion or spirituality; ‘media related factors’ when remission is considered possible through the aid of diverse media such as personal records, journals, conferences, the internet, television; and ‘treatment factors’ such as various biological or psychological approaches and interestingly alternative therapies.

Although people recognize the need for treatment, the notion of how this begins can be very broad. Motivation and stimulus to change can have several influxes of determination and start from an inner factor, a certain perception or insight, or from external factors, such as affective relationships or pregnancy. The idea of risk, the danger to one's health and, especially, physical complications or the risk of death seem to cause one, in these critical situations, to be in touch with reality in a way that triggers and promotes change. According to Vansteenkiste, and coworkers [34], motivation consists of a series of processes that make an individual move towards a specific objective. This is not about a personality trait, but rather a state that involves inner processes subject to change. Motivation is characterized by a dynamic process based on the transtheoretical model, developed by Prochaska and DiClemente [35]. This model describes the stages of behavioral change that an individual goes through in a non-linear way, whether in treatment or not. Ambiguity and reluctance to recover are important factors to be overcome [8].

Second, another type of competence needed for remission is empowerment, i.e. the development of the ability to put one's own life and identity in a new perspective. This takes into consideration the development of one's self-acceptance and the self and a sense of self-integration, a structure that can counterbalance the powerful mechanisms of the disease. These elements could consist of the perception of physical, psychological and spiritual values. Data from this study point to several factors that are involved in this manner: the capacity of self-observation, as a quality that is present or through spirituality; and the development of autonomy in relation to the family environment. Existing evidence suggests that religion and spirituality are important improvement factors in some clinical and mental disorders [21], [36], [37]. It is therefore interesting to investigate this area further.

Third, different types of media, especially in western societies, promote the cult of beauty, define body standards and establish types of behavior. The data found have provided surprising evidence that the media can have a clarifying and informative role, being capable of changing beliefs and types of behavior and thus contributing to remission. There is a widespread notion that AN patients tend to value their complaints and body shape and to enjoy participating in internet websites in order to promote and reinforce their symptoms and treatment resistance [38]. In addition, there is a preliminary study indicating that an internet discussion group was seen as helpful in the early stages of the disease [39].

The current study adds a new perspective in this area, considering the positive influence of various medias such as television, the internet, conferences and magazines and viewing them as beneficial for promoting help among AN sufferers. This is consistent with the idea that some media factors such as those we have detected in this study may have a positive role that contributes to AN remission. However, further research is needed to test this hypothesis [5].

Finally, despite the fact that AN is an old illness, effective treatment continues to elude clinicians. There are studies in this area, and none have identified clear empirical support for particular psychotherapeutic or pharmacological treatments. Most of the studies involving adolescents with AN suggest that family therapy is helpful in younger patients with an early onset and a short duration of illness [40]. However, **a**nother useful contribution to this study is the critical opinions AN patients have over treatments they have had. People recognize the role of qualified treatments (medical, psychotherapeutic, nutritional etc.) but many interviewees reported at the same time that the procedures provided partial or incomplete help. As a result treatments can and should be adjusted to each patient.

Remarkably our results have shown that non-authorized treatments, such as meditation and yoga, were very useful in the remission process [41]. We must emphasize however, that treating a difficult to treat patient may be uncomfortable for clinicians as decisions have to be made upon little empirical evidence or ethical barriers [42], [43]. Our findings, however, could be present in future epidemiological, clinical or experimental procedures to find alternative ethipathogenic or therapeutic factors.

Many women in our sample were uninformed about remission and were unprepared for it. Even when remission was achieved some respondents recognized the presence of remnants of the disease and the risks of falling ill again. This process is identified as a threat posed to one's life. Remission enables some habits and types of behavior to be changed, promoting participation in meaningful activities, such as social, affective and family functions. However, the analysis of interviews found that anorexia nervosa scars development, restricting previous and current activities. Due to this fact, researchers considered that, in certain cases, anorexia nervosa is characterized as a disease that can involve limitations and restrictions in the long term, enabling varying levels of adaptation despite remittance from the disorder [44].

Our findings are consistent with the growing body of the literature that suggest that remission includes behavioral, physical, psychological, emotional, environmental and social functioning [19], [27], [45], [46]. This study however expands this field by exploring not only other treatment aspects (psychotherapy, nutrition, pharmacotherapy), but life after remission and the recovery process. In addition our study sheds light on the importance of spiritual life and media factors as associated with remission. This unexpected and very interesting finding needs further investigation.

This study suggests three simple points for clinicians. First, knowing some core thematic information related to remission may help find alternatives for the treatment approach. Second, although alternative treatments are not a panacea, there is room for ethical and clinical considerations in some cases. Third, women with AN usually show reluctance and ambiguity relative to treatment and therefore it may be necessary to give them the opportunity to voice their concerns. Putting into consideration the use of information through several medias can open a door for change.

### Strengths and limitations

Some positive aspects can be emphasized as regards the performance of this study. First of all, the use of a qualitative methodology enabled us to distinguish factors, perceived as positive for the remission of AN, to be analyzed. Patients' clinical and narrative information was recorded in detail. The long follow-up period, the experience of patients with the disease, and the treatment enabled aspects, relevant in the construction of factors involved in remission, to be identified. Qualitative analysis allowed a coherent understanding of the process involved in remission. Finally, data enabled the formulation of hypotheses that can be used in future studies.

Various limitations can be considered when analyzing the results of the present study. To start with, participants brought information about the disease, treatments and other events throughout their lives. This procedure is subject to memory bias. In addition, differing abilities of communication and recording of memories should be pointed out. However, reducing researcher bias involved the system of analysis and the search for the maintenance of methodological rigor and field supervision as well as the consensual preparation of analytical categories. We did not ask participants about outcome parameters like BMI, diet and menstruation. However there is evidence that it is important to consider not only eating behavior and weight, but also psychological, emotional, and social elements as criteria for recovery [45]. Participants may have had contact with other sources of help and it is conceivable that this procedure might have, in part, contributed to remission. We had no control on that. The sample size was small but number of participants was determined by saturation. Finally information must be analyzed with caution, given the fact that the sample mostly consisted of women with a high level of education and income and a low rate of hospitalization.

### Future research

Although we did not interview the family members or the partners of patients, their points of view were constantly present in several interviews. If asked directly, they may have provided different and richer contributions. Further research is needed to fill this gap. A detailed assessment of alternative treatments is called for to determine to what extent these approaches are used and what impact, if any, they have on the women's AN. More work is needed to fully determine the role of media – such as the internet, television, conferences – on patients' AN. Further research could also give some insight into the clinicians' perspective on delivering tailored treatment approaches to women with AN and test the impact on outcome. The wide diversity of patients with AN, including the non-complete eating disorder not otherwise specified (EDNOS), calls for a more proactive coordination of care and of consistent strategies to address unmet needs.
